# Establishment of Cell Lines from Both Myeloma Bone Marrow and Plasmacytoma: SNU_MM1393_BM and SNU_MM1393_SC from a Single Patient

**DOI:** 10.1155/2014/510408

**Published:** 2014-08-12

**Authors:** Youngil Koh, Woo-June Jung, Kwang-Sung Ahn, Sung-Soo Yoon

**Affiliations:** ^1^Department of Internal Medicine, Seoul National University Hospital, 101 Daehak-ro, Jongno-Ku, Seoul 110-744, Republic of Korea; ^2^Cancer Research Institute, Seoul National University College of Medicine, 103 Daehak-ro, Jongno-Ku, Seoul 110-799, Republic of Korea; ^3^Functional Genome Institute, PDXen Biosystem Inc., 437 Dongil-ro, Kwangjin-Ku, Seoul 143-901, Republic of Korea

## Abstract

*Purpose.* We tried to establish clinically relevant human myeloma cell lines that can contribute to the understanding of multiple myeloma (MM). *Materials and Methods.* Mononuclear cells obtained from MM patient's bone marrow were injected via tail vein in an NRG/SCID mouse. Fourteen weeks after the injection, tumor developed at subcutis of the mouse. The engraftment of MM cells into mouse bone marrow (BM) was also observed. We separated and cultured cells from subcutis and BM. *Results.* After the separation and culture of cells from subcutis and BM, we established two cell lines originating from a single patient (SNU_MM1393_BM and SNU_MM1393_SC). Karyotype of the two newly established MM cell lines showed tetraploidy which is different from the karyotype of the patient (diploidy) indicating clonal evolution. In contrast to SNU_MM1393_BM, cell proliferation of SNU_MM1393_SC was IL-6 independent. SNU_MM1393_BM and SNU_MM1393_SC showed high degree of resistance against bortezomib compared to U266 cell line. SNU_MM1393_BM had the greater lethality compared to SNU_MM1393_SC. *Conclusion.* Two cell lines harboring different site tropisms established from a single patient showed differences in cytokine response and lethality. Our newly established cell lines could be used as a tool to understand the biology of multiple myeloma.

## 1. Introduction

Multiple genetic and microenvironmental changes [1] lead to the transformation of postgerminal center B cells into malignant neoplasm. Multiple myeloma is a malignant B cell disorder characterized by proliferation of atypical plasma cells in bone marrow [2] with or without the presence of monoclonal immunoglobulin protein in serum and/or urine [3]. Over the past decades, several effective treatment strategies have been developed for multiple myeloma [2]. These include high-dose chemotherapy supported with autologous peripheral blood stem cells, proteasome inhibitors [4], and immunomodulatory drugs such as thalidomide and lenalidomide [5]. However, despite these advances, multiple myeloma is still thought to be an incurable disease. And researchers are vigorously on the way to understand the biology of myeloma in order to improve the clinical outcome of myeloma patients.

By the way, multiple myeloma has correlation with plasmacytoma, which is a mass of plasma cells found outside of bone marrow [6] that needs medical intervention with radiotherapy [7] or chemotherapy. While multiple myeloma frequently accompanies plasmacytoma at the time of diagnosis, plasmacytoma precedes multiple myeloma in some cases. The disease entity called primary extramedullary plasmacytoma exists in 4% of plasma cell tumors [8, 9], and approximately 40–50% of patients with solitary plasmacytoma will develop multiple myeloma [10]. Hence, plasmacytoma is an early form or an accompanying disease of myeloma, and the data regarding the “clinical behavior” of plasmacytoma are quite accumulated. However, not much is known about the cellular biology of plasmacytoma per se. For example, cell lines established from plasmacytoma are not abundant with less than 10 cell lines with evident plasmacytoma available at ATCC (https://www.atcc.org/).

Many clinicians are curious about the adequate treatment strategy of plasmacytoma [11]. And these clinical problems can be answered with the study focusing on plasmacytoma. Considering the tropism of plasmacytoma, cell clone in bone marrow in myeloma and a cell clone in plasmacytoma would have difference. We think focusing on that difference is a key factor for understanding plasmacytoma biology.

For this aspect, we present, in this study, the establishment of two human multiple myeloma cell lines, called SNU_MM1393_BM and SNU_MM1393_SC from a patient with aggressive multiple myeloma using an animal model. SNU_MM1393_BM cell line is derived from bone marrow of a mouse, and SNU_MM1393_SC is derived from a subcutaneous plasmacytoma. Here, we characterized phenotypic, genetic, and functional properties of these cell lines. Also, we further investigated the response to cytokines and chemotherapeutic agents of these cell lines.

## 2. Materials and Methods

### 2.1. Case History

In February 2012, a 63-year-old male patient visited Seoul National University Hospital for back pain and tingling sense on the trunk below nipple. He was diagnosed as multiple myeloma with spinal cord compression due to osseous plasmacytoma on the third thoracic vertebrae. His disease stage was 3 by Durie-Salmon staging and 2 by International Staging System. Karyotype of this patient was normal, but fluorescent in situ hybridization (FISH) revealed that the disease had trisomy 9,* RB1* deletion, trisomy 1q,* IgH* rearrangement, and* IgH* 3 copies. His myeloma cell secreted monoclonal protein of immunoglobulin A, kappa chain. He received radiotherapy for vertebral plasmacytoma. After radiotherapy, four cycles of thalidomide/dexamethasone chemotherapy were given to the patient, which yielded in partial response (PR). Stem cell collection was performed in preparation for autologous stem cell transplantation after PR to thalidomide/dexamethasone chemotherapy. We used bone marrow cells at the time of stem cell collection for this experiment. The karyotype was sustained to be normal by the time of this stem cell collection. He is still in PR after autologous stem cell transplantation with high-dose melphalan conditioning with progression free survival time of 8.3 months. He did not receive either bortezomib or panobinostat.

### 2.2. Cell Culture and Establishment of Cell Line

Bone marrow specimens were obtained from a patient diagnosed with multiple myeloma under a protocol approved by the Seoul National University Hospital Institutional Review Board. Mononuclear cells were separated by Ficoll-Hypaque density sedimentation. Eight-week-old NRG/SCID mice were injected intravenously via the dorsal tail vein (i.v.) with 1 × 10^6^ mononuclear cells suspended in a total volume of 300-microliter PBS. After 14 weeks, bone marrow specimens were obtained from mice, and isolated bone marrow cells were cultured in RPMI-1640 medium (Gibco-BRL, Gaithersburg, MD, USA) supplemented with 10% heat-inactivated fetal bovine serum, penicillin (100 U/mL), and streptomycin (100 g/mL) (GIBCO, Grand Island, NY, USA). They were cultured in a highly humidified atmosphere of 5% CO_2_ and 95% air at 37°C. The medium was exchanged every 3-4 days depending on the rate of cell growth.

### 2.3. Histopathology and Cytogenetic Analysis

Histological sections of bone marrow from NOD/SCID mice were prepared and stained with haematoxylin and eosin (H&E) using standard methods. Cell morphology was examined using light microscopy.

Metaphase chromosome spreads from peripheral blood and the cell line were prepared and G-banded according to standard procedures. The karyotype was described according to the International System for Human Cytogenetic Nomenclature (ISCN) (2009) [12].

### 2.4. Cell Proliferation Assay

Cell proliferation assay was performed using Cell Counting Kit-8 (Dojindo Laboratories, Kumamoto, Japan) according to the manufacturer's instructions. Means and standard deviations were generated from three independent experiments. Absorbance values were normalized to the values obtained from control group to determine the value for % of survival.

### 2.5. Western Blot Analysis

The cells were treated with indicated reagents for the indicated time periods, washed once in ice-cold phosphate buffered saline (PBS), and resuspended in lysis buffer (20 mM MOPS (pH 7.0), 2 mM EGTA, 5 mM EDTA, 30 mM sodium fluoride, 60 mM b-glycerophosphate (pH 7.2), 20 mM sodium pyrophosphate, 1 mM sodium, orthovanadate, 1% Triton X-100, 1 mM PMSF, aprotinin, leupeptin, and pepstatin 1 mg/mL). The protein concentration of lysate was measured, 30 *μ*g of whole cell protein extracts was boiled for 5 min, and the proteins were resolved in 10% SDS-polyacrylamide gel electrophoresis and electrotransferred to polyvinylidene difluoride membranes (Millipore, Bedford, MA, USA). The membranes were blocked in Tris-buffered saline containing 0.05% Tween 20 and 5% nonfat dry milk for 1 h at room temperature and incubated with the appropriate primary antibody for 2 h. Immunoreactive proteins were detected using horseradish peroxidase-conjugated secondary antibody (Jackson ImmunoResearch Laboratories Inc., PA, USA) and enhanced chemiluminescence reagents (Amersham Pharmacia Biotech, Piscataway, NJ, USA).

### 2.6. FACS Analysis

Cells (1 × 10^6^ cells/mL) were incubated with 20 *μ*L of phycoerythrin-conjugated anti-CD138 and anti-CD45, respectively (Becton Dickinson, San Jose, CA, USA) for 30 min at 4°C, washed, and then fixed with 2% paraformaldehyde. Then, the samples were analyzed by FACSCalibur flow cytometer (Becton Dickinson) and inbuilt software.

### 2.7. Drug Response Evaluation

Cells were treated with various concentrations of bortezomib and panobinostat, respectively, for 72 hours. After the treatment, cell proliferation assay was performed to examine their growth inhibitory effects. As a control experiment, we used U266 cells and U266_SC cells.

### 2.8. Use of Commercially Available Cell Lines

We used commercially available cell lines which were bought from DSMZ (http://www.dsmz.de/) as a control experiment in our study. The used cell lines include U266, U266_SC, IM9, and RPMI8226 cell lines.

## 3. Results

### 3.1. Establishment and Cytogenetic Characterization of SNU_MM1393_BM Cell Line and SNU_MM1393_SC Cell Line

**Figure 1:**
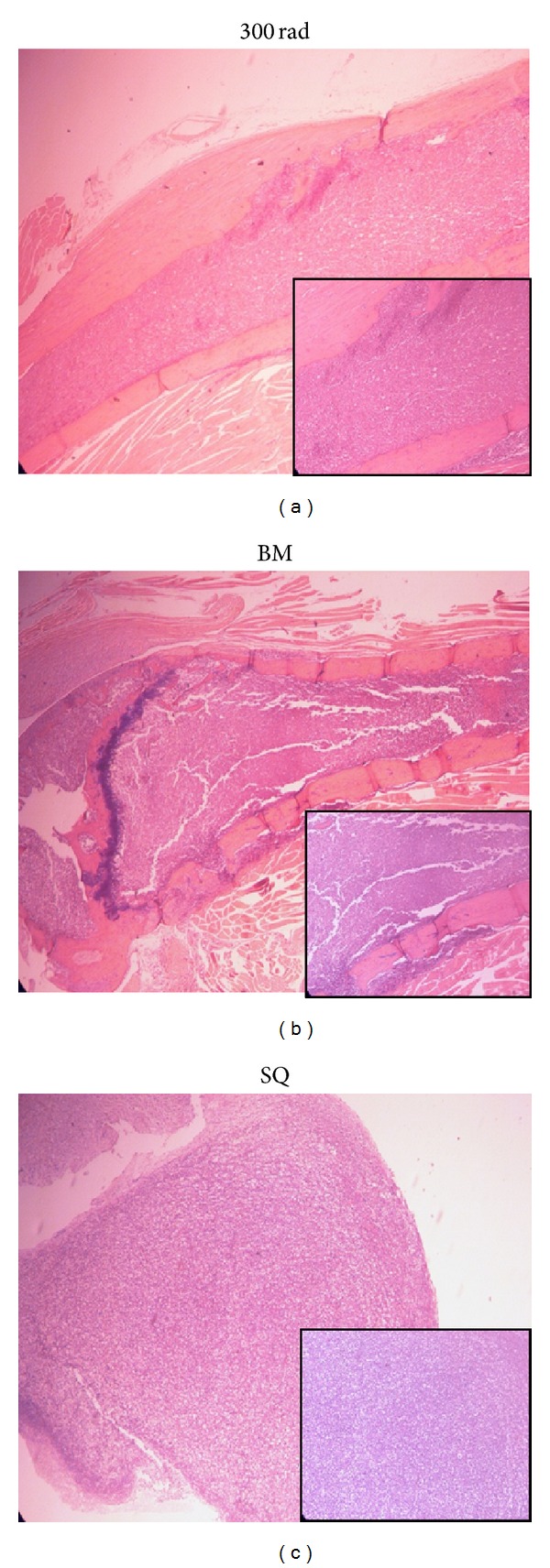
Representative appearances of histopathologic hematoxylin and eosin (H&E) staining of the irradiated bone marrow (a), bone marrow of the leg with cancer cell infiltration (b), and tumors at subcutis (c).

**Figure 2:**
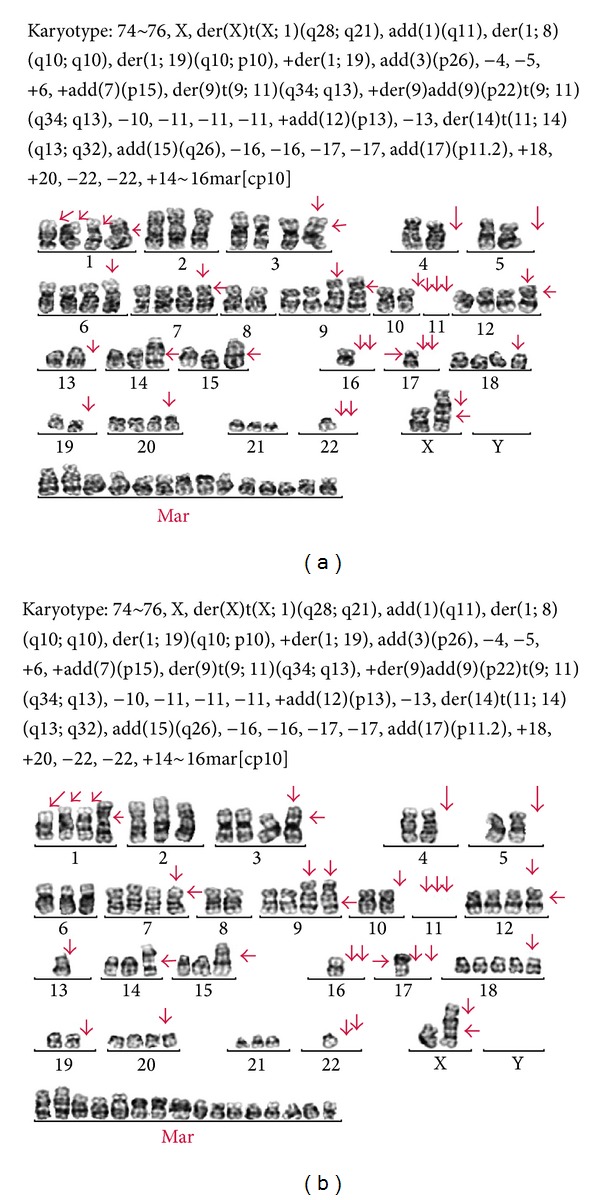
Karyotype of SNU_MM1393_BM (a) and SNU_MM1393_SC (b) cell line using G-banding.

Fourteen weeks after the injection of patient's mononuclear cell to NRG/SCID mouse, we found that myeloma cell grew at a subcutis. We also observed myeloma cells engrafted into bone marrow (H&E stain) (Figure 1). Myeloma cells obtained from both sites had undergone* ex vivo* culture and those cells successfully grew in RPMI-1680 with 10X FBS. They have reached over 20 population doublings through culture crisis and have been growing rapidly compared to the established myeloma cell lines. Doubling time was approximately 48 hours. A karyotype of the patient at diagnosis was displayed as normal which was in a diploidy. Interestingly, when cytogenetic analysis was performed for the newly established cell lines, the karyotype of SNU_MM1393_BM and that of SNU_MM1393_SC were near-tetraploidy (Figure 2). Hence, karyotypes of newly established multiple myeloma cell lines were different from those of original patient at diagnosis. Moreover, there was a difference in karyotypes between SNU_MM1393_BM and SNU_MM1393_SC. Chromosome 13 loss was noted in both cell lines, but there was a difference in copy numbers. That is, while SNU_MM1393_BM had 2 copies loss in chromosome 13, SNU_MM1393_SC cell line had 3 copies loss. Also, differences were noted in chromosome 6 and chromosome 18.

**Figure 3:**
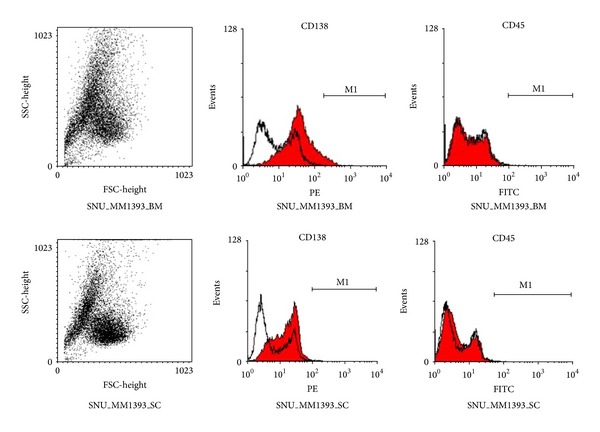
Flow cytometry analysis of SNU_MM1393_BM and SNU_MM1393_SC cell lines. Expression of CD138 and CD45 of both cell lines was analyzed using fluorescein-activated cell sorting (FACS).

As shown in Figure 3, the expression patterns of CD138 and CD45 by FACS analysis signified that both cell lines are of lymphoid origin. Also, light Giemsa stain revealed that the established cell lines have large, round nuclei, 1-2 nuclei/cell, and basophilic cytoplasm as previously mentioned [13].

### 3.2. IL-6 Mediated Cell Signaling Pathway Was Differentially Regulated between the Two Cell Lines

When we evaluated the cytokine response in these cell lines, differential response was noted between the two cell lines. We used cytokine interleukin-6 (IL-6) and soluble IL-6 receptor (sIL-6R), which is well known to regulate the biologic behaviors of myeloma cells in the progression of multiple myeloma. It is well known that sIL-6R potentiates the IL-6 mediated signaling [14]. Based on these, we treated myeloma cells with IL-6 and sIL-6R together to determine the activation of IL-6 mediated signaling pathway. Incubation of multiple myeloma cells with 5 nM IL-6, 25 nM sIL-6R, and combined treatment of IL-6 and sIL-6R (5 nM and 25 nM) for 30 min, respectively, was performed.

**Figure 4:**
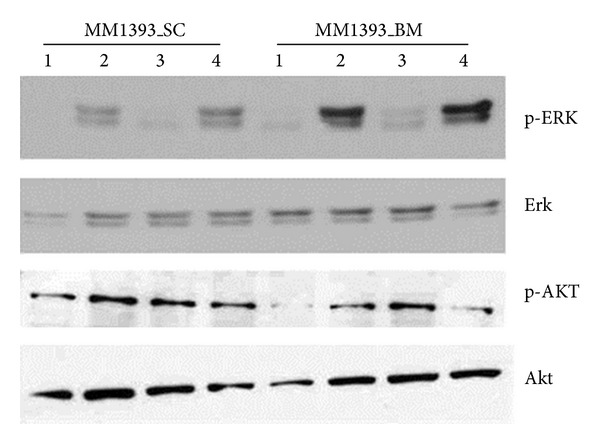
Western blot showing response to IL-6 in SNU_MM1393_BM and SNU_MM1393_SC. Whole cell lysates obtained from both cell lines were treated with IL-6, sIL-6R, and combined IL-6 and sIL-6R for 30 min (1: control, 2: IL-6 5 ng/mL, 3: sIL-6R 25 ng/mL, and 4: IL-6 5 ng/mL + sIL-6R 25 ng/mL).

In myeloma cells established from bone marrow of a mouse (SNU_MM1393_BM), phosphorylated form of Erk (p-ERK) was increased when treated with IL-6 only and combination of IL-6 and sIL-6R, but phosphorylated form of Akt (p-AKT) was not. On the other hand, in myeloma cells established from subcutaneous plasmacytoma (SNU_MM1393_SC), the induction of p-ERK was not as evident as in SNU_MM1393_BM. Increase in p-AKT was not evident in SNU_MM1393_SC either (Figure 4).

**Figure 5:**
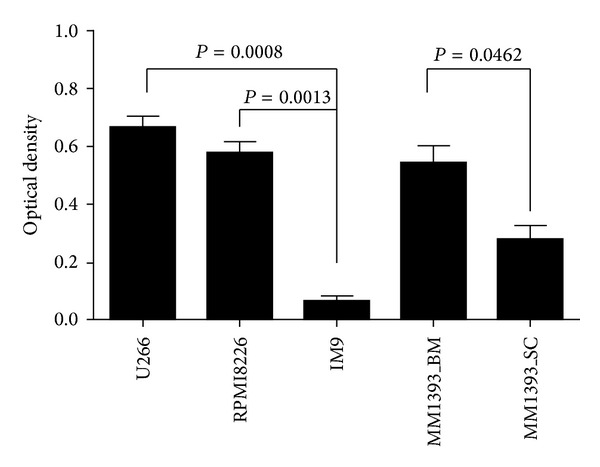
Production of IL-6 from SNU_MM1393_BM and SNU_MM1393_SC, U266, RPMI8226, and IM9.

Since IL-6 acts in an autocrine manner [15], we measured the amount of IL-6 in culture soup of U266, RPMI8226, IM9, SNU_MM1393_BM, and SNU_MM1393_SC, respectively, after those cells were cultured with 1% FBS for 72 hours. Result of SNU_MM1393_BM was similar to that of U266 and RPMI8226, suggesting that it is an IL-6 dependent cell line. However, SNU_MM1393_SC and IM9 released only a small amount of IL-6 (Figure 5). And any changes in the amount of sIL-6R in both cell lines were not observed.

### 3.3. Drug Response of Newly Established Cell Lines

**Figure 6:**
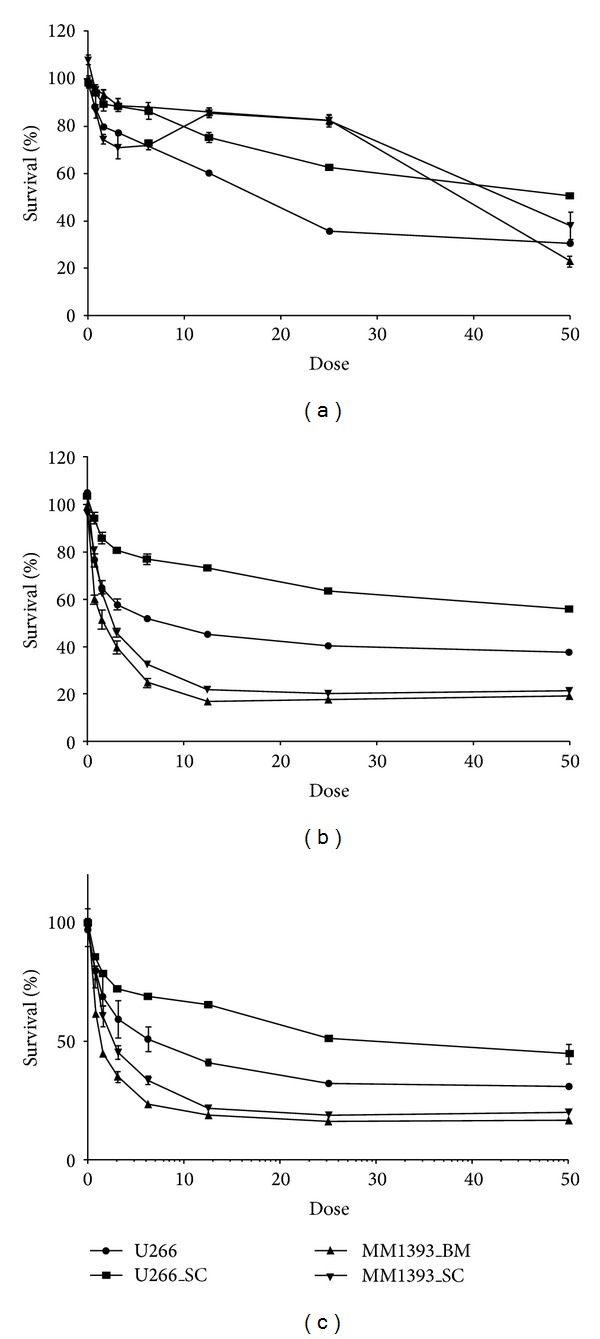
Growth inhibitory effect of bortezomib (a), panobinostat (b), and combination of bortezomib and panobinostat (c) in newly established MM cell lines. The data shows the mean ± standard error of three independent experiments.

We tested the cytotoxic effect of proteasome inhibitor bortezomib and pan-HDAC inhibitor panobinostat in these two cell lines. Both SNU_MM1393_SC and SNU_MM1393_BM were more sensitive to panobinostat when compared to U266 (*P* < 0.05). Meanwhile, SNU_MM1393_BM seemed to be more sensitive to panobinostat compared to SNU_MM1393_SC (*P* > 0.05). However, when compared to well-known myeloma cell lines (U266 and U266_SC), SNU_MM1393_SC and SNU_MM1393_BM were more resistant to bortezomib (*P* value <0.05) (Figure 6).

### 3.4. Determination of Tumorigenicity of the Established Two Cell Lines

To determine the tumorigenicity of these cells, we reinjected these* ex vivo* cultured cells via tail vein into NRG/SCID mouse. We used three kinds of cells: (1)* ex vivo* cultured myeloma cells of a patient's bone marrow, (2) cells from SNU_MM1393_BM, and (3) cells from SNU_MM1393_SC. When this was performed, we found tumor growth at both bone marrow and subcutis with* ex vivo* cultured myeloma cells. However, when cells from SNU_MM1393_BM were injected into a mouse, growth at subcutis was not noted. Only engraftment at bone marrow was observed. Lastly, when SNU_MM1393_SC cells were injected into a mouse, a mouse died in 7 weeks after injection. Tumor at subcutis was not noted at the time of death.

## 4. Discussion

It is known that the establishment of multiple myeloma cell line is notoriously difficult [16]. Here, we report two newly established cell lines from a single patient with multiple myeloma. Newly established cell lines secreted the kappa light chain, which was the similar to myeloma cells at diagnosis of our patient. Cell-surface markers of established cell lines exhibit positive expression of CD138, CD45, and CD34 as shown in multiple myeloma cells from a patient at diagnosis. Given these observations, we consider two newly established cell lines (SNU_MM1393_BM and SNU_MM1393_SC) that have originated from the donor patient's primary myeloma cells. Also, in a separate study, we performed whole exome sequencing of these two cell lines to reveal genetic difference between these two cell lines. In the study, genomic signature of these two cell lines was almost the same except for some single nucleotide variants and copy number variations, which implies that these two cell lines are from a single patient.

We observed several interesting findings during the establishment of these cell lines. First, there was a difference between patient's original myeloma cells and established cell lines. While patient's karyotype was a diploidy, karyotype of SNU_MM1393 was tetraploidy. This reflects a clonal evolution of a myeloma cell during cell line establishment. In fact, there are reports regarding the clonal evolution to near-tetraploidy both* in vivo* and* ex vivo*. As an* in vivo* report, Yuan et al. [17] reported a case of female patient who became lenalidomide refractory after one year of treatment with lenalidomide/steroid. The patient experienced clonal evolution to near-tetraploidy with chromosome 13 loss. As an* ex vivo* data, Balsas et al. [18] reported that RPMI8226 cell line which became resistant to bortezomib after serial cultivation evolved to near-tetraploidy. Hence, we assume that clonal evolution to near-tetraploidy is related to drug resistance and aggressive tumor biology. We assume that change of a host from human to NRG/SCID mouse in our experiment would act as a tremendous stress to myeloma cell, and the cell from our patient would evolve to near-tetraploidy. In fact, tetraploidy is frequently observed in myeloma patients at the time of diagnosis [19]. In a cohort at Seoul National University Hospital which consists of 80 myeloma patients, tetraploidy at diagnosis was noted in 3 patients. One interesting report by Koren-Michowitz et al. is that 13q deletion and IgH abnormalities which have a prognostic value in multiple myeloma might have some correlation with near-tetraploidy [20]. We think that tumor biology associated with near-tetraploidy needs further research.

From the above, we conjecture that aggressive myeloma cells might have been selected through cell line establishment using animal model. And, as expected, SNU_MM1393_SC had greater lethality than original myeloma cells of the patient. Mouse injected with SNU_MM1393_SC died within 7 weeks, while mouse injected with the original myeloma cells survived more than 14 weeks. One interesting finding in this experiment (regarding the tumorigenicity of established cell line) is that SNU_MM1393_BM was not as lethal as SNU_MM1393_SC. This coincides with the results that SNU_MM1393_SC showed more prominent resistance to bortezomib and SNU_MM1393_SC had more chromosome 13 loss which is a well-known negative prognostic marker in multiple myeloma [21]. Various experiments using these two cell lines are underway in our laboratory, and detailed clinicopathologic information including pharmacologic profile of these cell lines will be reported soon.

Second, there were differences between SNU_MM1393_BM and SNU_MM1393_SC in various aspects (Table 1). In an experiment regarding response to cytokine, these differences were dramatic. Various cytokines, especially IL-6 and sIL-6R, directly affect the biologic behaviors of multiple myeloma cells [14, 22], and the response to IL-6 in myeloma cells is very important. Because IL-6 is known to act in an autocrine manner [15], the induction of IL-6 and sIL-6R needs to be analyzed in each myeloma cell line. And, when this was analyzed, degree of IL-6 induction was different between SNU_MM1393_BM and SNU_MM1393_SC, with low IL-6 induction level in SNU_MM1393_SC. The similar results were found in coculture analysis. These findings suggested that myeloma cells growing at soft tissue were not dependent on IL-6 mediated cell signaling. And we think that aggressive biology of SNU_MM1393_SC is related to cell growth potential independent of IL-6 signaling. However, this conjecture needs further research for verification.

Lastly, interesting findings were when drug response was investigated in these cell lines. Most importantly, newly established cell lines showed relative resistance to bortezomib compared to U266. This is an unexpected phenomenon, because the myeloma cell of our patient is bortezomib naïve. Considering relative indolent clinical course of our patient, it is unusual that a myeloma clone of our patient is resistant to bortezomib. When used with dexamethasone, response rate of bortezomib in treatment-naïve myeloma patients is 88%. Hence, we conjecture that this resistance to bortezomib shown in our newly established cell line would originate from clonal evolution during cell line establishment. We suggest that biologic behaviors of myeloma cells could be altered in the course of clonal expansion and this alteration would contribute to the chemotherapeutic resistance.

Whether the above findings could be generalized to myeloma cells in bone marrow and plasmacytoma is questionable. Not many researches have been performed regarding the comparison of bone marrow myeloma cells and plasmacytoma, and we think our study results should be recognized as exploratory ones. Comparison with future researches focusing on plasmacytoma biology is necessary to adapt our findings to clinical practices. We are also willing to supply our established cell lines to other researchers for further studies.

## 5. Conclusion

Two cell lines harboring different site tropisms established from a single patient showed differences in cytokine response. Our newly established cell lines could be used as a tool to understand the biology of multiple myeloma and its chemotherapeutic responses.

## Figures and Tables

**Table 1:** Comparison of SNU_MM1393_BM and SNU_MM1393_SC cell lines.

	Chromosome 13 loss	IL-6 dependence	Bortezomib resistance	Panobinostat resistance	Plasmacytoma generation	Tumor lethality
Original tumor	None	Unknown	Unknown	Unknown	+	−
SNU_MM1393_BM	2 copies	+	+	−	−	−
SNU_MM1393_SC	3 copies	−	++	−	−	+

NA: not applicable.
